# Isotope Encoded Spatial Biology Identifies Amyloid Plaque-Age-Dependent Structural Maturation, Synaptic Loss, and Increased Toxicity

**DOI:** 10.21203/rs.3.rs-5829037/v1

**Published:** 2025-01-22

**Authors:** Jack I. Wood, Maciej Dulewicz, Junyue Ge, Katie Stringer, Alicja Szadziewska, Sneha Desai, Srinivas Koutarapu, Haady B. Hajar, Lydia Fenson, Kaj Blennow, Henrik Zetterberg, Damian M. Cummings, Jeffrey N. Savas, Frances A. Edwards, Jörg Hanrieder

**Affiliations:** 1 Department of Psychiatry and Neurochemistry, Sahlgrenska Academy at the University of Gothenburg, Mölndal Hospital, House V, S-431 80 Mölndal, Sweden; 2 Department of Neuroscience, Physiology and Pharmacology, University College London, Gower Street, London, United Kingdom; 3 Department of Pathology and Immunology, Washington University School of Medicine, Saint Louis, Missouri, USA; 4 Clinical Neurochemistry LaboratoryMemory Clinic, Sahlgrenska University Hospital, Mölndal Hospital, House V, S-431 80 Mölndal, Sweden; 5 Paris Brain Institute, ICM, Pitié-Salpêtrière Hospital, Sorbonne University, Paris, France; 6 Neurodegenerative Disorder Research Center, Division of Life Sciences and Medicine, and Department of Neurology, Institute on Aging and Brain Disorders, University of Science and Technology of China and First Affiliated Hospital of USTC, Hefei, P.R. China; 7 Department of Neurodegenerative Disease, Queen Square Institute of Neurology, University College London, Queens Square, WC1N 3BG London, United Kingdom; 8 UK Dementia Research Institute at UCL, London, UK; 9 Hong Kong Center for Neurodegenerative Diseases, Clear Water Bay, Hong Kong, PR China; 10 Wisconsin Alzheimer’s Disease Research Center, University of Wisconsin School of Medicine and Public Health, University of Wisconsin-Madison, Madison, WI, USA; 11 Department of Neurology, Feinberg School of Medicine, Northwestern University, Chicago, IL 60611, USA; 12 Department of Neuropsychiatry, Sahlgrenska University Hospital, Gothenburg, Sweden

## Abstract

Understanding how amyloid beta (Aβ) plaques form and progress to neurotoxicity in Alzheimer’s disease remains a significant challenge. This study aims to elucidate the processes involved in Aβ plaque formation and maturation using a knock-in Aβ mouse model (*App*^*NL-F/NL-F*^). By employing mass spectrometry imaging and stable isotope labeling, we timestamped Aβ plaques from their initial deposition, enabling the spatial tracking of plaque aging. Correlating single-plaque spatial transcriptomics with time since seeding, allowed us to track gene-expression changes specifically associated with plaque age, independent of chronological age of the mouse or disease severity. We found that plaque age, within sections from individual mice aged from 10 to 18 months, negatively correlates with synaptic gene expression. Further, correlation with hyperspectral confocal microscopy using structure-specific dyes revealed a positive link between plaque age and structural maturity, with older plaques identified as more compact and associated with significantly greater synapse loss and toxicity.

## Introduction

The mechanisms underlying Alzheimer’s disease (AD) pathogenesis are still not fully understood. AD pathology is characterized by extracellular Aβ plaques and intracellular hyperphosphorylated Tau tangles with dementia^1^. The prevailing model of AD pathogenesis is that changes in Aβ metabolism precipitate a damaging cascade upstream of tau pathology and eventual neurodegeneration^2^. The relevance of Aβ peptides in AD has seen a recent resurgence as Aβ targeting antibodies lecanemab and donanemab have seen positive outcomes in Phase 3 trials as well as FDA approval^3,4^. However, Aβ levels are elevated in AD brains years before synapse loss, circuit dysfunction, neurodegeneration, and impaired cognition^1^. It is therefore important to capture the disease at the earliest events of Aβ plaque formation. An understanding of Aβ pathogenesis is complicated by the heterogenous presentation of plaque pathology including cored plaques but also diffuse- as well as vascular plaques^5,6^. At a cellular level, Aβ has been found to alter synaptic vesicle dynamics^7^, where exo- and endocytosis machinery are already altered in the very early stages of Aβ pathology onset^8^ and the probability of transmitter release has been shown to be altered even before plaques can be detected^9,10^. Clinically, this is in line with the hypothesis that Aβ pathology precipitates several decades before cognitive symptoms occur. Most importantly, these early effects of Aβ aggregation, including synaptic changes, precede the development of mature Aβ plaques that can be detected by PET imaging or by CSF analysis^11–13^. Exactly how plaques develop over time, the extent of their diversity and their relation to toxicity or homeostatic response of surrounding neuronal circuits remains unclear^14^.

Novel developments within chemical imaging, such as mass spectrometry-based imaging (MSI)^15–17^ together with stable isotope labelling greatly increase our ability to study these events. Imaging of stable isotope labelling kinetics (iSILK) allows the delineation of plaque formation dynamics in a spatiotemporal manner while maintaining high molecular specificity^18^.

Herein, we make use of these developments and interface the iSILK approach with both hyperspectral microscopy and spatial transcriptomics. Importantly, the dynamic nature of this approach allows us to track precipitating plaque pathology in the *App*^*NL-F/NL-F*^(*App*^*NLF*^) AD mouse model, which mirrors the age-associated and gradual plaque development observed in human Alzheimer’s disease. We demonstrate that not only do plaques cause local synaptic damage as has been previously^19^ demonstrated but individual plaques cause increasing synaptic damage as they persist and mature in the brain. Together this novel spatial biology approach provides a detailed picture of Aβ-aggregation, plaque formation, and maturation in concert with the associated cellular and molecular response in and around individual plaques at scales not previously possible.

## Results

### iSILK delineates spatial and structural patterns of plaque formation and maturation

**Fig 1.**
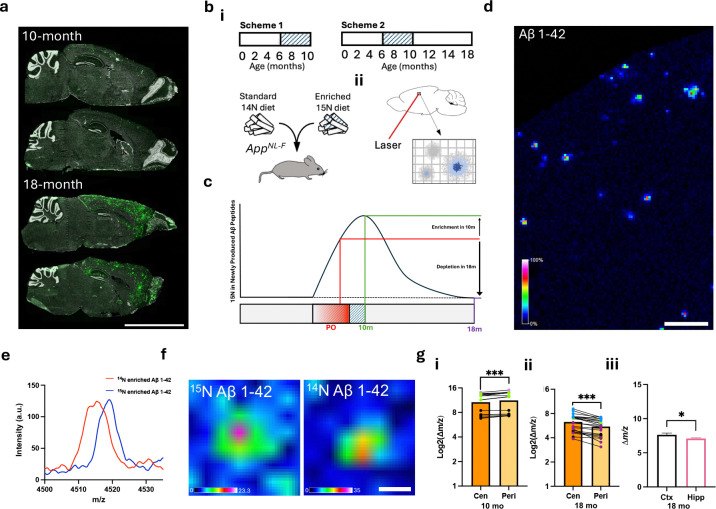
iSILK delineates spatial and structural patterns of plaque formation and maturation. (A) Representative sagittal images of plaque load (green) of 10- and 18-month-old APP^NL-F^ mice (B) Pulse-chase iSILK paradigm (i) feeding schemes for ^15^N enriched diet, pulse period between 6–10 months of age. PO indicates plaque onset, with initial hippocampal plaques appearing between 6–9 months (iii) MALDI MSI scheme detailing the collection of Aβ peptides from defined rasters. (C) Predicted ^15^N enrichment of newly synthesized Aβ peptides over the course of the pulse-chase feeding paradigm. (D) MALDI MSI produced single ion image of Aβ1–42 in a section of temporal cortex. Scale bar 200μm (E) Example spectra from MALDI MSI showing the Aβ1–42 m/z peak. (F) Representative MALDI MSI image of ^15^N and ^14^N enriched plaques. Scale bar 20μm (G) Aβ1–42 mass analysis comparing (i) the plaque centre (Cen) vs. the periphery (Peri) in 10-month-old mice, (ii) in 18-month-old mice, and (iii) differences between cortex and hippocampus. (i, ii) Linear Mixed Model, animal indicated by point color, (iii) Paired t-test. Data presented as mean ± SEM. Significance levels: *** P<0.001, **P<0.01; *P<0.05.

Initial amyloid aggregation and subsequent plaque formation are dynamic processes, and difficult to capture whilst tracking across chronological ageing. We previously demonstrated the potential of stable isotope labelling and MSI to assess relative timing of Aβ plaque formation within individual *App*^*NL-G-F*^ mice^18^. While these initial experiments were key for establishing the technique and its feasibility, there are limitations with respect to the *App*^*NL-G-F*^ model whereby the Arctic mutation of APP alters the chemical properties of Aβ leading to an increased aggregation propensity not seen in sporadic AD. This results in a rapid plaque onset with a pronounced formation of cored deposits, lacking the range of plaque isoforms seen in the human disease^20^. Therefore, by building on the previous findings from *App*^*NL-G-F*^ mice, we aimed to follow Aβ aggregation in the more slowly progressing plaque pathology of *App*^*NL-F*^ mice. This model provides a more physiological presentation of amyloid aggregation due to a gradual increase in Aβ. Although rare plaques have been reported to appear as early as 6 months ^20^, we reliably detect the first plaques at 9 months. Thereafter plaque load and size along with microglial response is increasing steadily to 18months and reach a plateau by 24 months^10,20^ (Fig. 1A). This gradual increase in plaque pathology with age more closely resembles the human disease.

For this study, *App*^*NL-F*^ mice were labelled with ^15^N spirulina diet from age 6–10 months (PULSE period, 4 months, Fig. 1Bi) and either culled immediately at 10 months old (Fig. 1Bi, Scheme 1), or after an 8-month CHASE (washout) at 18 months old (Fig. 1Bi, Scheme 2). Here, *App*^*NL-F*^ mice at 10 and 18 months of age represent early and established stages of plaque pathology, respectively (Fig. 1A).

For scheme 1, continuous labeling was used to identify whether the labelling scheme was implemented in time to ensure that secreted Aβ peptides were labelled prior to extracellular deposition and precipitation in plaques^20^. MALDI MSI was then used to determine stable isotope enrichment in plaque-associated, deposited Aβ peptides in situ (Fig. 1Bii and 1C). This approach enables us to image plaques by spatially measuring Aβ1–42 species (Fig. 1D) and correlating the isotope enrichment of the deposited Aβ peptides with the different plaque structures such as the center and periphery, thereby investigating the temporal development of these features during plaque formation.

At 10 months of age, the sparse deposition of Aβ plaques in the cortex contained solely Aβ1–42. Importantly, it was observed that all Aβ1–42 peptides detected showed ^15^N incorporation (Fig. S1). This ensured that the onset of the labelling scheme at 6 months old was sufficient to incorporate the ^15^N label into APP prior to plaque pathology onset, observed at 9 months. After establishing the feasibility of the iSILK approach in *App*^*NL-F*^ mice, we then set out to interrogate deposition dynamics within and across plaques and brain regions.

First, to address the question of intra-plaque heterogeneity (center vs. periphery), the relative ^15^N content of these plaque structures was evaluated. For this, we isolated plaque ROI spectral data of hippocampal and cortical deposits (Fig. 1). We compared the isotopologue signature of plaque-associated Aβ1–42 that encodes the degree of labelling, as indicated by the shift in mass (Fig. 1). For an unbiased, quantitative comparison of label incorporation, we performed curve analytics of the isotope envelope for a plaque-associated signal that in turn encodes the degree of label incorporation. Here, to estimate the shift in m/z value as caused by the ^15^N label and hence the difference in label content, we compared the centroid of the curve fitted to the Aβ1–42 isotopologue pattern, attributed to the comparably low mass resolution in linear mode (Fig. 1E). These tradeoffs with linear mode were needed to ensure sensitive peptide detection at high spatial resolution.

Based on the labelling scheme, this shift in the isotopologue signature indicates earlier or later deposition. Here, plaques in 10-month-old *App*^*NL-F*^ mice (PULSE age 6–10 months, no CHASE) displayed lower ^15^N content for Aβ1–42 in the center compared with the periphery (Fig. 1C, 1F and 1Gi, P<0.01). This suggests that previously deposited Aβ1–42 species have less ^15^N at the center, while later secreted Aβ1–42 deposits at the periphery. In 18-month-old animals (Scheme 2, PULSE age 6–10 months, 8-month CHASE), the pattern was reversed due to the significant deposition of unlabeled Aβ1–42 during the CHASE period (Fig. 1C and 1Gii, P<0.05).

Both experiments suggest indeed that in precipitating amyloid plaque pathology in *App*^*NL-F*^ mice the center of the plaque represents the first deposited Aβ. Over time, this initial structure of the plaque gets gradually compacted into a fibrilized core upon homogenous plaque-wide deposition of less aggregated Aβ during the plaque growth phase.

While these first analyses identified deposition patterns within plaques, iSILK further allows us to delineate plaque formation sequences across different brain regions. This is important as it allows for a description of how plaque pathology spreads throughout the brain and in what sequence it affects vulnerable brain regions. Similar to the plaque heterogeneity analyses, isotope incorporation was determined from the isotopologue patterns of Aβ 1–42 in plaque features detected in the cortex and hippocampus as described above. The results show that the degree of isotope content, as expressed as the centroid of the fitted isotopologue distribution curve, was higher in cortical plaques compared to hippocampal plaques (Fig. 1Giii). This indicates that the cortex is the primary location of initial plaque formation, which is in line with immunohistochemical studies described for this model as well as previous iSILK data obtained for *App*^*NL-G-F*^ mice^18^.

### Amyloid plaque maturation is characterized by continuous fibrilization with age

**Fig 2.**
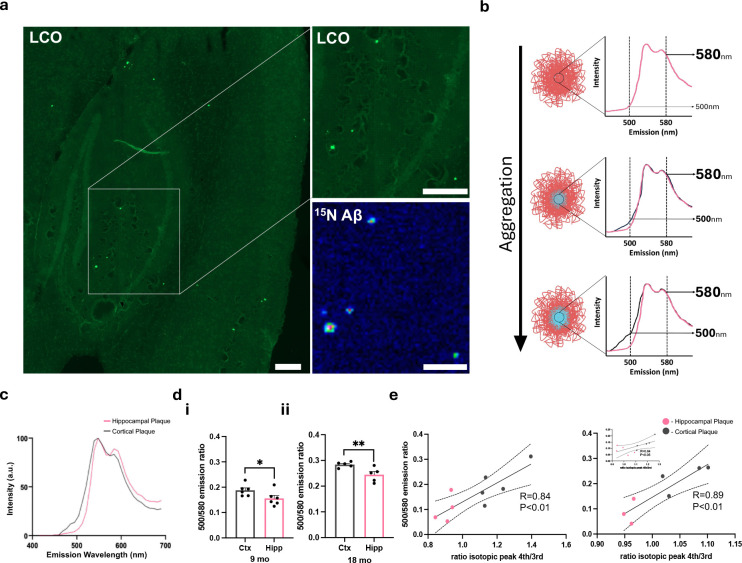
Amyloid plaque maturation is characterized by continuous fibrilization with age. (A) Representative images demonstrating plaque detection using LCO staining and MALDI MSI. Scale bar 200μm (B) Schematic illustrating increasing 500nm intensity resulting from q-HFTAA binding as plaques aggregate. (C) Averaged mass spectra comparing hippocampal and cortical plaques. (D) Analysis of the emission ratio, with the 500nm q-HFTAA peak against the 580nm h-HFTAA peak, in cortex vs. hippocampus for 9-month-old (i) and 18-month-old (ii) mice. Paired t-test (E) Pearsons’s correlation analysis of the 500/580nm ratio with the Aβ1–42 isotopic peak ratio for three 18-month-old mice. Data presented as mean ± SEM. Significance levels: **P<0.01; *P<0.05.

Since we identified that the center of the deposits observed at both 10 and 18 months represents the oldest part of the plaque, we also aimed to investigate the structural maturation of plaques as they age in addition to changes in gene expression. Therefore, we integrated the iSILK MSI experiments with functional fluorescent microscopy using structure-specific amyloid probes: luminescent conjugated oligothiophene (LCO)^21^, allowing for the identification of morphologically heterogenous amyloid structures within the Aβ plaques (.. 2A). This is enabled by the difference in affinity of the two LCO probes, q- FTAA and h-FTAA, towards amyloid aggregates. Specifically, q-FTAA preferentially binds to mature and compact beta-pleated aggregates, while h-FTAA binds to less compact, yet still beta-pleated aggregates. Due to their different emission profiles, the LCO probes can be spatially delineated using hyperspectral fluorescent microscopy. Here, the ratio of the LCO maxima (500nm for q- FTAA / 580nm for h-FTAA) is used to express preferential binding of either of the two LCO probes used, whereby an increase in 500nm intensity is indicative of increased q-FTAA binding and therefore, increased structural maturity of the amyloid fibrils (Fig. 2B). As only few plaques were detected by MALDI in the 10-month-old animals (Scheme 1), of which all were cortical, we focused on 18-month-old *App*^*NL-F*^ animals (Scheme 2, Fig. 1Bi) and compared plaques in the cortex to those in the hippocampus. Similar to the iSILK data showing that cortical plaques are older than those in the hippocampus, our results demonstrate that cortical plaques also exhibit higher 500/580 nm ratios at their centers, suggesting that they are more aggregated (Fig. 2C). To further test this hypothesis, we performed a single plaque correlation analysis of correlative iSILK/LCO data collected from plaques detected across two sequential tissue sections. The results indeed, showed a positive correlation (R 0.63–0.89, p<0.05, Fig. 2E) of label incorporation and LCO emission ratio (Fig. 2D). Together these data are the first of its kind showing a direct correlation of the time course of individual plaques with biophysical measures of amyloid aggregation within individual animals. These results confirm that in these mice, plaques appear to continuously mature in terms of amyloid fibrillization at the plaque core.

### iSILK-guided spatial transcriptomics shows changes in synaptic-, metabolic-, and immune-associated gene expression with plaque age.

**Fig. 3.**
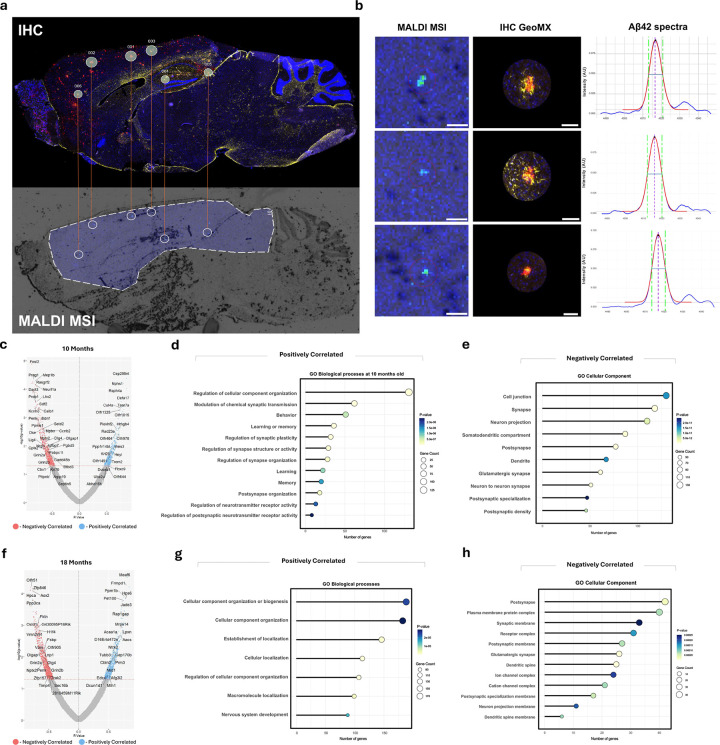
iSILK guided spatial transcriptomics shows changes in synaptic-, metabolic-, and immune-associated gene expression with plaque age. (A) Matching of Aβ plaques on consecutive sections for immunohistochemistry (IHC) and MALDI MSI imaging. (B) Aβ signals of single plaques across MALDI MSI and IHC images, including m/z spectra with peak and Full Width Half Maximum (FWHM). Scale bar: 100μm. (C) Volcano plot for gene expression correlations with increasing plaque age in 10-month-old *App*^*NL-F*^ mice (Scheme 1). (D, E) Gene ontology analyses of negaively correlated genes in 10-month-old mice, presented in lollipop plots. (F) Volcano plot for gene expression correlations with increasing plaque age in 18-month-old APP^NL-F^ mice. (Scheme 2). (G, H) Gene ontology analyses of positively (G) and negatively (H) correlated genes in 18-month-old mice, presented in lillipop plots.

Utilizing the iSILK technique to track plaque age, we conducted single plaque spatial transcriptomics to monitor gene expression changes associated with plaque age, independent of the mouse’s chronological age. Spatial transcriptomics was selected over other sequencing techniques due to its ability to target plaque-specific gene expression changes, offering a more spatially resolved technique for AD pathology-associated alterations compared to the more commonly used RNA sequencing methods^22^ (Fig. S2). The regions of interest (ROIs) chosen on the spatial transcriptomics platform were selected by identifying double positive (MALDI and IHC) plaques across two serial sections, with the MALDI MSI images imported and overlaid onto the IHC image for precise mapping (Fig. 3A, 3B, and Table S1). As plaques were selected across serial sections, the plaques analyzed were all large and therefore plaque size showed no significant correlation with plaque age (P = 0.25, r=0.29).

To identify associations between the plaque age (MALDI iSILK) and whole transcriptome-wide gene expression, we performed correlation analysis across all plaques between peak centroid and quantile normalized counts for all genes. Volcano plots for both 10-month (Fig. 3C) and 18-month-old (Fig. 3F) mice demonstrate significant up- and down-regulation of genes associated with plaque age.

In 10-month-old animals, functional annotation of correlated genes revealed that (Scheme 1) older plaques are associated with increased expression of genes related to immune regulation and microglial response (e.g. Trem2, IL6, IL1rn, Defa17, Fcgr2b, Axl) (Fig 3C, Tab. S2). In contrast, synaptic genes (*Nptx2, Nptxr, Grin2a, Grin2b, Nrgn*) were negatively associated with plaque age (Fig. 3D,E, Tab. S2).

In 18-month-old animals (Scheme 2), the gene ontology of correlated genes shows that older plaques are linked to an increase in expression of genes involved in metabolic processes (*Atp5a1*, *Atp5k*, *Cox7a2*, *Coa6*, *Coq7*) and channel activity (*Kcnj2*, *Kcna2, Scn1a*, *Scn2b* Fig. 3G, Tab. S2) compared to younger plaques in the same animal. In contrast, increasing age of plaques correlated with a concomitant decrease in genes predominantly associated with postsynapses and synaptic membrane (*Shisa6*, *Chrna9*, *Drd1*, *Nptx2, Gria1, Grin2b, Calb1;* Fig. 3H, Tab. S2).

Using synaptic hub genes^23^ and disease-associated genes^24^, we compared expression profiles between previously generated spatial transcriptomics data and RNA-seq results from bulk hippocampal tissue analysis^25^ (Fig. S2). While s patial transcriptomics effectively captured expression changes associated with Alzheimer’s pathology, bulk RNAseq was less sensitive in detecting these differences. Additionally, incorporating plaque age, as enabled by iSILK, reveals further changes related to plaque maturity that are overlooked when only considering plaques at a given chronological age^25^ (Fig. S2). This underscores the significant advantages of spatial transcriptomics in addressing diseases with strong spatial characteristics, such as AD.

In summary, at 10 months, older plaques are associated with increased immune-related gene expression, while at 18 months, they show higher transcription of metabolism-related genes. In both age groups, plaque aging is consistently linked to a decline in synapse-related gene expression. This confirms, as previously observed that synapses are damaged when exposed to plaques. However, it additionally suggests that it is not only initial exposure that causes damage but that the toxicity continues to increase over the time of exposure to a particular plaque, independent of the age of the animal. Alternatively, the individual plaque may become more toxic as it remains for a longer period in the tissue. In contrast it seems that the immune response to the plaque is an acute response to early exposure as plaques start to build up but does not change greatly as the plaque remains in the tissue for longer periods. However longer exposure, interacting with the age of the animal and/or the total burden of plaque, results in an increasing metabolic load, possibly in response to increasing toxicity of the older plaques.

### Differences in Synapse Loss and Toxicity Revealed by LCO-Defined Plaque Types.

**Fig 4.**
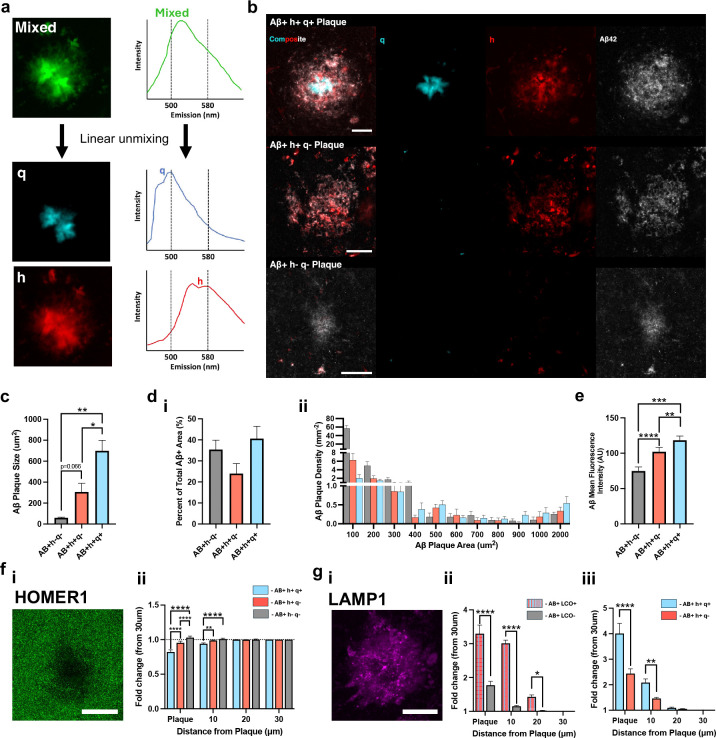
Differences in Synapse Loss and Toxicity Revealed by LCO-Defined Plaque Types. (A) Schematic illustrating the linear unmixing of mixed LCO signal into isolated q-HFTAA and h-HFTAA channels. (B) Representative images of three LCO defined plaque types. (C) Average area covered by individual plaques. (D) (i) Percentage of total Aβ area covered by each plaque types. (ii) Histogram distribution of plaque areas. (E) (Average Aβ fluorescence intensity of plaques. (F) (i) Image illustrating HOMER1 loss associated with plaques. (ii) Analysis of protein fluorescence intensity relative to LCO-defined plaque types. (G) (i) Image showing increased LAMP1 expression at plaques. (ii, iii) Quantification of protein fluorescence intensity relative to LCO-defined plaque types. Statistical Analysis: (C, D, E) One-way paired ANOVA, n=6. (F, G) Two-way repeated measures ANOVA. (F: n=6, G(i): n=7, G(ii): n=4). Data in panels (C, D, E) are plotted as paired data, while panels (F, G) data are presented as mean ± SEM. Post-hoc Tukey’s test corrections applied. Significance levels: ****P<0.0001; ***P<0.001; **P<0.01; *P<0.05. Scale bar 20μm.

As amyloid aggregation / LCO probe binding is a factor of plaque age, we adapted our hyperspectral imaging approach to categorize amyloid plaques depending on the presence of q-FTAA and h-FTAA, representing different stages of plaque maturation. For this, we used linear unmixing, of the hyperspectral LCO data utilizing reference spectra of pure q-FTAA and h-FTAA to separate the probes into distinct channels (Fig. 4A). Additionally, an Aβ42 antibody served as a general Aβ marker. We focused exclusively on 18-month-old animals, as the 10-month-old group presented too few hippocampal plaques to yield a sufficient dataset. We identified three populations of structurally distinct plaque types depending on the positivity of the LCO probes: Aβ+h+q+, Aβ+h+q−, and Aβ+h−q−, here arranged in descending order of aggregation maturity (Fig. 4B). In the paired LCO/iSILK results above, we established that continuous plaque aging is associated with increased q+ staining i.e. amyloid fibrilization (Fig. 3E). This suggests that Aβ+q+h+, Aβ+q+h−, and Aβ+q−h− represent distinct stages of plaque maturation. Therefore, this static imaging approach allows study of plaque ages dissected from chronological age within the same animal using fluorescent microscopy-based correlated LCO imaging and immunohistochemistry within the same section.

On average, Aβ+h+q+ plaques were the largest in area accounting for 41% of Aβ-positive regions in the hippocampus, followed by Aβ+h+q− plaques, which occupied 24%. Of note, many small (<100mm^2^), LCO-negative (Aβ+h−q−) plaques were detected in the hippocampus of 18-month-old animals, which constituted 35% of the plaque-positive area (Fig. 4C & 4D). Those small immature plaques showed low Aβ intensity (Fig. 4E) and are difficult to capture in MALDI MSI, particularly spanning across two consecutive 12mm sections for correlative MALDI MSI/GeoMX or MALDI/LCO experiments, limiting these analyses to LCO pos. plaques. Moreover, when examining these plaque types over the lifespan of the mice, we observed that Aβ+h+q− and Aβ+h+q+ plaques together make up 90% of the Aβ-positive area in the hippocampus of younger, 9-month-old *App*^*NL-F*^ mice. By 14 months, the proportion of Aβ-positive area occupied by Aβ+h−q− plaques rose to 26% (53% of plaques by number), increasing further to 36% (78% of plaques by number) by 18 months (Fig. S3). This indicates that as the mice age, the number of plaques seeded without a fibrillar core is increase.

Together these findings support the notion that in this mouse model at pathology onset, plaques form as small cores, which continuously mature and fibrilize, respectively, through progressing amyloid deposition. We consequently focus on Aβ+h+q+ that are continuously detected through all stages of progressing plaque pathology. Considering the observation above that increasing plaque age is associated with changes in transcription of synaptic genes (Fig. 3), we measured synapse loss and toxicity surrounding the plaque types using antibodies against HOMER1 and LAMP1 respectively. Synapse loss correlated with increased plaque aggregation, with Aβ+h+q+ plaques demonstrating the greatest reduction in HOMER1 signal (Fig. 4F). This corresponded to higher levels of dystrophic neurites (LAMP1) around more aggregated plaque types (Fig. 4G).

## Discussion

Most studies attempting to understand the development of AD and neurodegeneration primarily consider factors such as chronological age and pathological severity. However, a detailed understanding of AD development still lacks a single plaque evolution-related perspective. For instance, it is unclear whether newly formed plaques in the same individual exert a different influence on surrounding tissues compared to older plaques. Furthermore, it remains an important question in AD research why Aβ plaques have a poor correlation with cognitive decline despite the amyloid hypothesis suggesting it as the disease trigger^26^. This gap in understanding is further emphasized by a population of people who live dementia-free lives but upon post-mortem have a brain populated with plaques (CU-AP individuals, Murray and Dickson, 2014; Serrano-Pozo et al., 2011). Interestingly, the brains of these patients are characterized by an abundance of diffuse plaques, suggested to be an immature state that exerts little toxicity to its surroundings. These findings suggest a close relationship between plaque age, structural maturity, and neurotoxicity. In this study, using a novel multiomic iSILK-driven approach, we demonstrate this by showing that in older mice (18mo), older plaques are associated with higher structural maturity than newly formed plaques, which is in turn associated with greater neurotoxicity and more extensive loss of synapses.

Traditional bulk sequencing methods required mouse models of Alzheimer’s disease to develop exaggerated levels of plaque pathology in order to minimize non-pathological tissue to capture plaque-induced transcriptomic changes^27,28^. Therefore, more disease-relevant Alzheimer’s disease models such as *App*^*NL-F*^ mice, that develop plaques gradually, with onset in older ages, and without APP overproduction were unable to produce sufficient plaque loads to influence bulk sequencing results^10,29,30^ (Fig. S2). While our previous work, along with other studies, has leveraged recent advancements in spatial transcriptomics to demonstrate plaque-specific alterations in gene expression^25,27,28,31,32^ these studies often combine plaque regions and therefore do not consider the heterogeneity of plaque types and maturity. This raises questions about the differential effects of plaque heterogeneity on surrounding tissue. For the first time, we have integrated spatial transcriptomics with a metabolic labelling and spatial mass spectrometry imaging paradigm (iSILK MALDI MSI) to provide insights into how gene expression evolves in conjunction with plaque maturation at the single plaque level. We observed a negative correlation between synaptic-associated genes and plaque age at both 10 months (Scheme 1) and 18 months (Scheme 2), indicating that while the loss of synaptic function or synaptic puncta is associated with increasing plaque age, this relationship stands at both early and late chronological stages. Thus plaques are increasingly toxic to synapses as a result of continued presence in the tissue which is independent of the age of the animal. Moreover, the plaque age-associated decrease in synaptic genes is in line with SILK proteomic experiments in mice showing impaired turnover of synaptic proteins^8^.

Seemingly, in 10-month-old mice, we managed to capture the initial immune response and increasing immune proliferation from the precipitation of the very first plaques. Surprisingly this increase in immune activity associated genes with increasing plaque was not seen in 18-month-old animals^33^. However, this does not necessarily indicate a diminished response of microglia, instead, it may reflect that microglial genes are equally upregulated across plaque ages in older animals because the heavier plaque load^25^. In contrast, at 18 months, we saw an increase in metabolic processes-associated genes with increasing plaque load. This potentially reveals that increasing toxicity of older, cored plaques results in increasing metabolic demand on the surrounding cells or possibly toxicity leading to mitochondrial dysfunction and resulting compensation.

The observed downregulation of Dlg4, Dlgap1, Grin2a and Grin2b genes with age of plaque in mice of both ages (10 and 18 months) indicates that the presence and maturation of plaques has a persistent negative effect on synaptic gene expression. This is consistent with the well-documented role of amyloid-beta in synaptic dysfunction, which is an early and critical feature of AD. In addition, the reduced expression of NMDA receptor subunits (Grin2a and Grin2b) has profound implications for neuronal function. The NMDA receptor is crucial for synaptic plasticity and its downregulation can impair learning and memory processes (Fig. 3D). Reductions in genes related to postsynaptic density further exacerbate this dysfunction by destabilizing postsynaptic density and impairing excitatory synaptic signaling.

Recent advances in amyloid imaging have introduced structural-specific dyes, facilitating the easy categorization of plaque types through hyperspectral imaging and linear unmixing^34–36^. This method offers an unbiased, more efficient and high-throughput alternative to previous techniques that relied on morphological examination by pathologists. Interestingly, we have identified three distinct populations of amyloid plaques in *App*^*NL-F*^ mice, characterized by their unique structural isoforms. These populations were: Aβ+h+q+, Aβ+h+q−, and Aβ+h−q−, likely corresponding to the morphologically defined dense cored, fibrillar, and diffuse plaques respectively^37^. Our findings indicate that in older *App*^*NL-F*^ mice, the majority of deposits exhibit a diffuse and non-pleated structure (Aβ+h−q− / diffuse populations), which induces lower LAMP1 levels and HOMER1 loss compared to more compact and larger LCO positive plaques. This finding of increased synapse loss with increasing structural maturity is in line with previous findings in humans on morphologically- and thioflavin-defined plaques^19,38,39^ and also aligns with findings in amyloid-positive CU-AP individuals, where diffuse plaques exhibit low levels of neurotoxicity^40,41^. Furthermore, the finding of increased neurotoxicity with increasing aggregation is also consistent with studies showing that a higher propensity for Aβ aggregation and the presence of dense cored plaques are associated with increased progression of clinical dementia^42–44^.

The process of precipitating plaque pathology is difficult to capture with conventional, steady state imaging techniques, particularly in comparably late-onset mouse models like *App*^*NL*-F^. Dynamic imaging approaches such as chemical or genetic *in vivo* labelling provided important insight, though, despite being *in vivo*, lack chemical specificity and/or temporal span ^33^. Using the iSILK-based approach employed here for dynamic imaging of amyloid peptide deposition, our results from 10-month-old (Scheme 1) *App*^*NL*-F^ mice indicate that a plaque center forms first as a small dense core which is however less fibrilized at the beginning and gradually compacted upon plaque maturation (Fig. 1 & S1). This is also supported by the investigation of structural plaque isoforms over chronological age showing ~90% of initial depositions (9-month-old *App*^*NL*-F^) in the hippocampus are already fibrilized (Fig. S3). We have seen similar results in a previous iSILK study on the *App*^*NL-G-F*^ model suggesting that plaques initially precipitate as small dense deposits ^18^. Indeed, the results show that plaques continue to fibrilize as we see a positive correlation between q-hFTAA binding and increasing plaque age. This indicates a dynamic maturation process whereby pre-fibrillar Aβ (hFTAA+) species progress to a more mature state at the center of a plaque. This is of interest as a chronological, static imaging study on plaque maturation in *App*^*NL-F*^ showed no difference in q/h emission across age, though a large variety of plaque maturation stages was observed across plaques within an age group ^45^.

Delineating the plaque age irrespective of chronological age, as presented here, facilitates the identification of plaque age specific maturation, highlighting the potential of the dynamic imaging method described here. Surprisingly, we also note a large increase in the presence of non-pleated (LCO-negative) plaques with chronological age. Here, at 18 months, ~80% of plaques (by number) were LCO-negative though, on average, very small (<100mm^2^). This could suggest that these small, latterly formed plaques do not deposit through initial core formation. This discrepancy could be explained by the mechanism of plaque compaction and seeding potentially driven by microglial activity ^46–49^. For instance, in older mice, the exceptionally high concentrations of Aβ42 could saturate the normal amyloid seeding and compaction processes. This hypothesis is further supported by studies indicating that in the absence of microglia, amyloid structures tend to be less compact ^50^. A limitation of the current study is that the small, LCO-negative plaques were not captured across two consecutive sections of the iSILK guides transcriptomics experiments, posing a central challenge that should be at the center of future investigations.

Together we present a conceptual and technical innovative study using a novel spatial biology paradigm to detail the earliest events of plaque deposition through plaque maturation. This suggests that these plaques continuously fibrilize with age and that the oldest, most fibrilized plaques show the highest level of toxicity towards associated synapses, which is an accumulated phenomena due to a consequence of continuous presence, growth and interaction with synapses over time.

The spatial and temporal resolution along with chemical precision achieved with the iSILK guided spatial biology approach, particularly with respect to the age of the AD mice studied, exceeds steady state technologies and consequently provides a dynamic dimension that would otherwise not be discernible.

## Materials and Methods

### Animal Experiment

All procedures and experiments on mice were performed at UCL with local ethical approval (06/05/2016) and in agreement with guidelines of the Institutional Animal Care and Use Committee (IACUC) and the Animals (Scientific Procedures) Act 1986. Male and Female APP knock-in mice (*APP*^*NL-F*^) carrying humanized Aβ sequence, along with the Swedish mutation (KM670/671NL) on exon 16 and the Beyreuther/Iberian mutations (I716F) on exon 17 were used in the study ^20^. Transnetyx (Cordova, TN, USA) genotyping services were used to determine the presence of the knock-in genes in breeders.

### Tissue Extraction

Animals were decapitated with the brain rapidly extracted on ice. For the metabolically labelled mice, one hemisphere was snap-frozen directly after isolation using liquid nitrogen (− 150°C)-cooled isopentane. For non-metabolically labelled mice, one hemisphere was snap-frozen directly after isolation on dry ice. In both cases the other hemisphere was drop fixed in 10% formalin (4% PFA) at 4°C overnight. Formalin was subsequently washed out and replaced with 30% sucrose, 0.02% sodium azide in phosphate-buffered saline (PBS) solution for long term storage at 4°C.

### Tissue Sectioning

For correlative GeoMx/ MSI experiments, consecutive, sagittal cryosections were collected at 10μm (slide #A, GeoMx) and 12 μm (slide #B, MSI) from fresh frozen brain tissue on a cryostat microtome (Leica CM 1520, Leica Biosystems, Nussloch, Germany) at −18°C. Sections were thaw mounted on SuperFrost Plus Slides for the GeoMx DSP platform, with consecutive sections mounted on conductive indium tin oxide (ITO) glass slides (Bruker Daltonics, Bremen, Germany) for MALDI-MSI. All tissue was stored at −80°C.

PFA-fixed brain tissue, collected for time course plaque staining and immunohistochemistry, was sectioned transverse to the long axis of the hippocampus at 30 μm using a frozen sledge microtome (Leica). Free-floating sections were collected and stored in 0.02% sodium azide in PBS at 4°C.

### Reagents

**Table:** 

Reagent	Source

MouseExpress (15N, 98%) mouse feed kit (15N/14N)	Cambridge Isotope Laboratories (Andover, MA, USA)
Acetone (Ac)	Fisher Scientific (Hampton, NH, USA)
Acetonitrile (ACN)	Fisher Scientific (Hampton, NH, USA)
Absolute ethanol (EtOH)	Fisher Scientific (Hampton, NH, USA)
Methanol (MeOH)	Fisher Scientific (Hampton, NH, USA)
Glacial Acetic Acid	VWR Chemicals (Radnor, PA, USA)
Paraformaldehyde (PFA)	Agar Scientific (Stansted, Essex, United Kingdom)
Glutaraldehyde	Agar Scientific (Stansted, Essex, United Kingdom)
Sodium cacodylate buffer	Agar Scientific (Stansted, Essex, United Kingdom)
Osmium tetroxide	Agar Scientific (Stansted, Essex, United Kingdom)
Agar 100 resin	Agar Scientific (Stansted, Essex, United Kingdom)
Formic acid (FA)	Honeywell Fluka, Germany
Trifluoroacetic acid (TFA)	Honeywell Fluka, Germany
2,5-Dihydroxyacetophenone (2,5-DHAP)	Sigma-Aldrich (St. Louis, MO, USA)
TissueTek optimal cutting temperature (OCT) compound	Sakura Finetek (AJ Alphen aan den Rijn, Netherlands)
Indium tin oxide (ITO)-coated conductive glass slides	Bruker Daltonics (Bremen, Germany)
Peptide calibration standard	Bruker Daltonics (Bremen, Germany)
Protein calibration standard	Bruker Daltonics (Bremen, Germany)
PELCO copper grids	Ted Pella (Redding, CA, USA).

### Matrix deposition for MALDI-MSI

Fresh frozen tissue sections on ITO-coated glass slides were fixed in 100% ethanol for 60 seconds, followed by 70% ethanol for 30 seconds. Lipids were removed by immersing the sections in Carnoy’s solution (6:3:1 ethanol/chloroform/acetic acid) for 110 seconds, followed by subsequent washes in 100% ethanol for 15 seconds, 0.2% trifluoroacetic acid in water for 60 seconds, and 100% ethanol for 15 seconds. The tissues then underwent peptide retrieval using formic acid vapor for 20 minutes. To facilitate peptide ionization and desorption, a matrix solution (15 mg/ml 2,5-dihydroxyacetophenone in 70% acetonitrile, 2% acetic acid, 2% trifluoroacetic acid) was applied using a TM sprayer (HTX Technologies, NC, USA) and recrystallized in 5% methanol vapor at 85°C for 3 minutes.

### MALDI-MSI Data Acquisition

MALDI-MSI was performed using a MALDI-time-of-flight (TOF) instrument (RapifleX, Bruker Daltonics) equipped with a scanning Smartbeam 3D laser. The acquisition was conducted at a pixel size of 10 μm with 200 shots per pixel at 90% power with a shot frequency of 10 kHz Spectra were collected within the mass range of 1500–6000 m/z in linear positive mode due to higher sensitivity at high spatial resolution (mass resolution: m/Δm=1000 (FWHM) at m/z 4515). A second set of data for correlative LCO microscopy was collected at 20 μm with 200 shots per pixel at 90% power with a shot frequency of 10 kHz in reflector mode (mass resolution: m/Δm=15000 (FWHM) at m/z 4512.3). The system was externally calibrated by spotting a mixture of peptide standard II and protein standard I (Bruker Daltonics). Total ion current normalization for each ROI was performed using the FlexImaging 5.1 software (Bruker Daltonics). Binning analysis, including the calculation of peak width and amplitudes per average spectra, was conducted using the peak analyzer function in Origin (v.8.1; OriginLab, USA). We implemented a robust pipeline to detect, model, and characterize peaks from MALDI-MSI spectra, optimizing accuracy through an asymmetric Gaussian fitting approach. First, local maxima were identified by filtering for intensities higher than their immediate neighbors, followed by selecting the main peak based on maximum intensity. A fitting range, extending symmetrically around the main peak, was established to include relevant peak data while excluding noise. Baseline correction was applied within this range to ensure precise intensity measurements, with negative values set to zero. Initial parameters for the asymmetric Gaussian model, including peak amplitude, center (m/z value), and asymmetric widths for the left and right flanks of the peak, were estimated directly from the data. Model fitting was performed using a nonlinear least squares method with bounded constraints to ensure realistic parameter estimation. The asymmetric Gaussian model accounted for natural irregularities in peak shapes, providing high fidelity in capturing peak characteristics. Key metrics such as the Full Width at Half Maximum (FWHM) and also goodness of fit (R2 > 0.95) was derived from the fitted curve, offering detailed insights into the peak shape and spread. Based on the peak analysis performed pipeline, the FWHM values for each ROI at 10 and 18 months were extracted for correlation analysis with gene expression from GeoMX AOI.

### Spatial Transcriptomics

#### Slide Preparation:

Slides were processed following the standardized protocol for fresh frozen samples for analysis at the GeoMx Digital Spatial Profiler (REF). The 10 μm cryosections were fixed in 10% formalin followed by ascending concentrations of EtOH. Slides were then immersed in 1X Tris EDTA at 100°C for 15 minutes in a pressure cooker. After washing in PBS, RNA targets were exposed to 1ug/μl of Proteinase K in PBS at 37°C for 15 minutes. Tissues were post-fixed in 10% formalin for 5 minutes at RT followed by 2* 5-minute washes in NBF Stop Buffer at RT. Tissues were subsequently incubated in whole transcriptome RNA probes overnight in a hybridization chamber held at 37°C. Off-target probes were washed out in 2* Stringent Solution or 25 minutes at RT. Nonspecific binding was blocked using Buffer W (Nanostring propriety blocking buffer) followed by incubation in conjugated antibodies in Buffer W for 2 hours at RT (mouse anti-GFAP Alexa-Fluor 488 conjugate (Invitrogen, #53–9892-82), mouse anti- Aβ40/42 Alexa Fluor 594 conjugate (Nanostring, #121301306). After which, nuclei were counterstained for 1 hour at RT (SYTO 13, #S7575). After a final wash in 2x SSC buffer, slides were placed into the GeoMx machine.

#### ROI Selection:

MALDI MSI images of consecutive sections were overlayed on the fluorescent image output from the GeoMx. Plaques spanning both sections were identified, and a circular region of interest (ROI) three times the radius of the plaque was centered around the plaque’s midpoint. All probes regardless of cell type were extracted per ROI.

#### Library preparation and readout:

DNA barcode oligomers were aspirated into well plates whereby the aspirate from one plaque corresponds to one well. The well plates were then dried and rehydrated in 10μl nuclease-free water and PCR cycled with GeoMx SeqCode master mix and SeqCode primers (Nanostring Technologies). Finally, the PCR product was purified with AMPure beads. Plates were subsequently sequenced on an Illumina NextSeq2000. FASTQ files were converted to *.dcc format with the GeoMx NGS Pipeline GUI.

### MALDI MSI - GeoMx Data Analysis

The Quality control (QC) analysis for NGS and Biological Probe QC was conducted utilizing the default GeoMX DSP parameters (GeoMX NGS Pipeline 2.3.4). All further analyses were carried out in R [R Core Team, Vienna Austria, https://www.R-project.org/]. Probes were excluded from analysis if counts were too low or failed the Grubbs outlier test according to NanoString guidelines. Nine ROIs in the 10-month-old group and six ROIs in the 18-month-old group were excluded from further analysis due to ambiguous spot morphology >1 plaque or very low intensity in MALDI results (Fig. 3A). After data filtering, the geometric mean was calculated for targets in each AOI. QC results were compared with those from the standard NanoString pipeline, showing generally consistent outcomes in outlier detection. Any minor discrepancies are likely attributable to slight differences in the test parameters. Quantile normalization was applied using the normalize: quantiles function provided by the *preprocessCore* R package. The centroid of each Aβ 1–42 peak from the MALDI imaging results was used as an estimate for 15N incorporation and was calculated for the peak area outlined between the local intersection with the fitted baseline^18^ (Fig. 3B).

Pearson’s correlation was run between quantile normalized gene expression and centroid MALDI MSI centroid data for Aβ 1–42. The correlation results were divided into positive and negative, considering age (10-month-old positively/negatively and 18-month-old positively/negatively correlated). Gene over-representation enrichment (ORA) analysis was performed for each group separately, including the three different Gene Ontology (GO) terms related to Biological Processes (BP), Cellular Component (CC) and Molecular Functions (MF). For the ORA analysis, we applied a p-value and q-value cutoff of 0.05, using the entire gene set as a background. This analysis was conducted using the two R packages, *geneset* and *genekitr*. Data visualizations were carried out using in-house functions and modification of already existing functions, as a part of packages: GOplot, StringDB tidyr msigdbr.

### Immunohistochemistry and LCO staining

Free-floating 30 μm sections underwent antigen retrieval in 10 mM PH9 Tris-EDTA for 30 minutes at 80°C. Subsequently, sections were permeabilized in 0.3%Triton X-100 in PBS (PBST) for 3×10 minutes at RT before non-specific binding was blocked in 3% goat serum in PBST (Blocking solution) for 1 hour at RT. Sections were incubated with primary antibodies diluted in blocking serum at 4°C overnight (1:500 mouse anti-Aβ (6E10, #SIG-39320), 1:500 rabbit anti-Aβ (#700254), 1:200 chicken anti-HOMER1 (#160001), 1:500 rat anti-LAMP1 (#ab25245)). Sections were washed in PBST 3* 10 minutes at RT followed by incubation in secondary antibodies diluted in blocking solution for 2 hours at RT (1:500 goat anti-mouse AF594 (#A11032), 1:500 goat anti-rabbit AF594 (#A11037), 1:500 goat anti-chicken AF647 (#A-21449), 1:500 Donkey anti-rat AF594 (#A-21209)). Nuclei were counterstained with 4ʹ,6-diamidino-2- phenylindole (#ab228549) for 5 minutes at RT.

For structural amyloid analysis, tissues were stained with two luminescent conjugated oligothiophene (LCO) dyes, tetra- (q) and heptameric (h−) formyl thiophene acid (FTAA). For linear unmixing analysis after antibody staining, sections were subsequently incubated for 30 minutes at RT with 3μM q-FTAA and 1μM h-FTAA.

For LCO MALDI correlation analysis, cryo-sections were fixed sequentially in a gradient concentration of ice-cold 95%, 70% ethanol, and 1× phosphate-buffered saline (PBS) at room temperature followed by incubations in q-FTAA, 2.4μM in Milli-Q water and h-FTAA, 0.77μM in Milli-Q water) in the dark for 25 minutes.

### Fluorescent Microscopy

All imaging was performed on the Zeiss LSM780 confocal microscope. The imaging of double LCO-stained tissues was performed in hyperspectral mode using the 32-channel GaAsP spectral detector. The dyes were stimulated using a 35nW, 458nm argon laser. For LCO correlation analysis, continuous emission spectra were obtained in lambda mode. For plaque-type classification, linear unmixing mode utilized reference spectra of q-FTAA and h-FTAA to isolate the corresponding signals as individual channels.

For LCO correlation analysis individual amyloid plaques were randomly selected and images as z-stacks (20 stacks per plaque, 1-μm apart) under a 20X air objective. The 500:580 emission ratios were collected using Zen Black 2.3 software.

In the linear unmixing analysis, LAMP1, Aβ, HOMER1, q-FTAA and h-FTAA images were taken as z-stacked (3μm apart) tile scans of whole hippocampus using a 20X air objective. LAMP1, Aβ and HOMER1 were imaged in channel mode using constant light, gain and exposure settings matched to the fluorophore.

### LCO and MALDI MSI correlation analysis

Z-stacked images of individual plaques were analyzed in lambda mode, selecting the plane that showed the most pronounced 500nm shift for further examination. Within this plane, a small circle, just a few pixels in diameter, was placed on the area of the plaque exhibiting the highest 500nm shift, indicating the core. At this location, intensities at both 500nm and 580nm were recorded for analysis.

For the MALDI imaging data acquired in reflector mode, regions of interest (ROIs) were annotated, and total ion current normalized average spectra of the annotated ROIs were exported as .csv files in flexImaging. This was followed by a binning analysis for data reduction. Here, all ROI data were imported into Origin (v 8.1 OriginLab, Northampton, MA, USA) and the Peak Analyzer function in Origin was used to determine the isotopic peak area of Aβ 1–42 within each ROI spectrum. The ratio of the 4th to the 3rd isotopologue peak areas of Aβ 1–42 was used to assess label incorporation corresponding to the relative age of the plaque. Correlation analysis was then conducted between the 500/580 nm ratio in the hyperspectral data and the 4th/3rd isotopic peak area ratio of the same plaque detected in the corresponding MALDI imaging data. 7–10 plaques per animal from 3 animals were selected for this correlation analysis. Correlation statistics for all genes whole transcriptome can be searched at: https://hanriederlab.shinyapps.io/PlaqueAgeTranscriptomics/.

### IHC protein intensity towards plaque type

Image analysis was carried out using a set of custom macros for ImageJ. In short, the Z-stack tile scans were projected to a single plane based on standard deviation. Regions of interest (ROIs) were then drawn around each hippocampus to restrict further analysis to this area. Plaques were subsequently thresholded and categorized based on the positivity of both Aβ and LCO dyes. Radiating rings from each plaque were created at 10 μm increments extending up to 30 μm with any overlapping areas removed from the analysis. Mean grey values for each protein of interest at each increment were averaged per plaque type for each hippocampal image. 2–3 hippocampal images were averaged for each animal. Due to technical difficulties, LAMP1 analysis was conducted in two stages: The first stage involved assessing LAMP1 in plaques that were both Aβ and LCO positive (specifically h-FTAA only) and in plaques that were Aβ positive but LCO-negative. The second stage focused on plaques positive for both h-FTAA and q-FTAA, as well as plaques positive for h-FTAA but negative for q-FTAA. In contrast, HOMER1 analysis with Aβ, h-FTAA, and q-FTAA staining, was completed in a single batch.

### Statistical Analysis

Statistical analysis was performed in either GraphPad Prism 9 or RStudio. Post hoc analysis using Sidak’s correction was applied only when a statistically significant interaction was detected. For the analysis of center versus periphery (Fig. 1), a linear mixed-effects model was employed to account for paired measurements within plaques and to account for variability between biological samples. In all statistical tests biological replicates were considered appropriately to avoid pseudoreplication.

## Supplementary Material

Supplement 1

## Data Availability

All data needed to evaluate the conclusions in the paper are present in the paper and/or the Supplementary Materials. The transcriptomics results are accessible at: https://hanriederlab.shinyapps.io/PlaqueAgeTranscriptomics/

## References

[1] Long J. M. & Holtzman D. M. Alzheimer Disease: An Update on Pathobiology and Treatment Strategies. Cell 179, 312–339, doi:10.1016/j.cell.2019.09.001 (2019). 31564456 PMC6778042

[2] Busche M. A. & Hyman B. T. Synergy between amyloid-beta and tau in Alzheimer’s disease. Nat Neurosci 23, 1183–1193, doi:10.1038/s41593-020-0687-6 (2020). 32778792 PMC11831977

[3] van Dyck C. H. Lecanemab in Early Alzheimer’s Disease. N Engl J Med 388, 9–21, doi:10.1056/NEJMoa2212948 (2023). 36449413

[4] Sims J. R. Donanemab in Early Symptomatic Alzheimer Disease: The TRAILBLAZER-ALZ 2 Randomized Clinical Trial. JAMA 330, 512–527, doi:10.1001/jama.2023.13239 (2023). 37459141 PMC10352931

[5] Rohr D. Label-free vibrational imaging of different Abeta plaque types in Alzheimer’s disease reveals sequential events in plaque development. Acta Neuropathol Commun 8, 222, doi:10.1186/s40478-020-01091-5 (2020). 33308303 PMC7733282

[6] Koutarapu S. Chemical signatures delineate heterogeneous amyloid plaque populations across the Alzheimer’s disease spectrum. bioRxiv, doi:10.1101/2024.06.03.596890 (2024).

[7] Cirrito J. R. Endocytosis is required for synaptic activity-dependent release of amyloid-beta in vivo. Neuron 58, 42–51, doi:10.1016/j.neuron.2008.02.003 (2008). 18400162 PMC2390913

[8] Hark T. J. Pulse-Chase Proteomics of the App Knockin Mouse Models of Alzheimer’s Disease Reveals that Synaptic Dysfunction Originates in Presynaptic Terminals. Cell Syst 12, 141–158 e149, doi:10.1016/j.cels.2020.11.007 (2021). 33326751 PMC7897324

[9] Cummings D. M. First effects of rising amyloid-beta in transgenic mouse brain: synaptic transmission and gene expression. Brain 138, 1992–2004, doi:10.1093/brain/awv127 (2015). 25981962 PMC4572488

[10] Benitez D. P. Knock-in models related to Alzheimer’s disease: synaptic transmission, plaques and the role of microglia. Mol Neurodegener 16, 47, doi:10.1186/s13024-021-00457-0 (2021). 34266459 PMC8281661

[11] Cairns N. J. Absence of Pittsburgh compound B detection of cerebral amyloid beta in a patient with clinical, cognitive, and cerebrospinal fluid markers of Alzheimer disease: a case report. Arch Neurol 66, 1557–1562, doi:10.1001/archneurol.2009.279 (2009). 20008664 PMC2796200

[12] Ikonomovic M. D. Early AD pathology in a [C-11]PiB-negative case: a PiB-amyloid imaging, biochemical, and immunohistochemical study. Acta Neuropathol 123, 433–447, doi:10.1007/s00401-012-0943-2 (2012). 22271153 PMC3383058

[13] Ikonomovic M. D. Post-mortem correlates of in vivo PiB-PET amyloid imaging in a typical case of Alzheimer’s disease. Brain 131, 1630–1645, doi:10.1093/brain/awn016 (2008). 18339640 PMC2408940

[14] De Strooper B. & Karran E. The Cellular Phase of Alzheimer’s Disease. Cell 164, 603–615, doi:10.1016/j.cell.2015.12.056 (2016). 26871627

[15] Michno W., Wehrli P. M., Blennow K., Zetterberg H. & Hanrieder J. Molecular imaging mass spectrometry for probing protein dynamics in neurodegenerative disease pathology. J Neurochem 151, 488–506, doi:10.1111/jnc.14559 (2019). 30040875

[16] Michno W. Chemical imaging of evolving amyloid plaque pathology and associated Aβ peptide aggregation in a transgenic mouse model of Alzheimer’s disease. J Neurochem 152, 602–616, doi:10.1111/jnc.14888 (2020). 31605538

[17] Michno W. Chemical traits of cerebral amyloid angiopathy in familial British-, Danish-, and non-Alzheimer’s dementias. J Neurochem 163, 233–246, doi:10.1111/jnc.15694 (2022). 36102248 PMC9828067

[18] Michno W. Following spatial Abeta aggregation dynamics in evolving Alzheimer’s disease pathology by imaging stable isotope labeling kinetics. Sci Adv 7, eabg4855, doi:10.1126/sciadv.abg4855 (2021). 34134980 PMC8208724

[19] Koffie R. M. Oligomeric amyloid beta associates with postsynaptic densities and correlates with excitatory synapse loss near senile plaques. Proc Natl Acad Sci U S A 106, 4012–4017, doi:10.1073/pnas.0811698106 (2009). 19228947 PMC2656196

[20] Saito T. Single App knock-in mouse models of Alzheimer’s disease. Nat Neurosci 17, 661–663, doi:10.1038/nn.3697 (2014). 24728269

[21] Nilsson K. P. Imaging distinct conformational states of amyloid-beta fibrils in Alzheimer’s disease using novel luminescent probes. ACS Chem Biol 2, 553–560, doi:10.1021/cb700116u (2007). 17672509

[22] Smith K. D., Prince D. K., MacDonald J. W., Bammler T. K. & Akilesh S. Challenges and Opportunities for the Clinical Translation of Spatial Transcriptomics Technologies. Glomerular Dis 4, 49–63, doi:10.1159/000538344 (2024). 38600956 PMC11006413

[23] Williams J. B., Cao Q. & Yan Z. Transcriptomic analysis of human brains with Alzheimer’s disease reveals the altered expression of synaptic genes linked to cognitive deficits. Brain Commun 3, fcab123, doi:10.1093/braincomms/fcab123 (2021). 34423299 PMC8374979

[24] Habib N. Disease-associated astrocytes in Alzheimer’s disease and aging. Nat Neurosci 23, 701–706, doi:10.1038/s41593-020-0624-8 (2020). 32341542 PMC9262034

[25] Wood J. I. Plaque contact and unimpaired Trem2 is required for the microglial response to amyloid pathology. Cell Rep 41, 111686, doi:10.1016/j.celrep.2022.111686 (2022). 36417868

[26] Morris G. P., Clark I. A. & Vissel B. Inconsistencies and controversies surrounding the amyloid hypothesis of Alzheimer’s disease. Acta Neuropathol Commun 2, 135, doi:10.1186/s40478-014-0135-5 (2014). 25231068 PMC4207354

[27] Chen W. T. Spatial Transcriptomics and In Situ Sequencing to Study Alzheimer’s Disease. Cell 182, 976–991 e919, doi:10.1016/j.cell.2020.06.038 (2020). 32702314

[28] Mallach A. Microglia-astrocyte crosstalk in the amyloid plaque niche of an Alzheimer’s disease mouse model, as revealed by spatial transcriptomics. Cell Rep 43, 114216, doi:10.1016/j.celrep.2024.114216 (2024). 38819990

[29] Sasaguri H. APP mouse models for Alzheimer’s disease preclinical studies. EMBO J 36, 2473–2487, doi:10.15252/embj.201797397 (2017). 28768718 PMC5579350

[30] Ghosal K. Alzheimer’s disease-like pathological features in transgenic mice expressing the APP intracellular domain. Proc Natl Acad Sci U S A 106, 18367–18372, doi:10.1073/pnas.0907652106 (2009). 19837693 PMC2762847

[31] Choi H. Spatiotemporal characterization of glial cell activation in an Alzheimer’s disease model by spatially resolved transcriptomics. Exp Mol Med 55, 2564–2575, doi:10.1038/s12276-023-01123-9 (2023). 38036733 PMC10767047

[32] Matarin M. A genome-wide gene-expression analysis and database in transgenic mice during development of amyloid or tau pathology. Cell Rep 10, 633–644, doi:10.1016/j.celrep.2014.12.041 (2015). 25620700

[33] Meyer-Luehmann M. Rapid appearance and local toxicity of amyloid-beta plaques in a mouse model of Alzheimer’s disease. Nature 451, 720–724, doi:10.1038/nature06616 (2008). 18256671 PMC3264491

[34] Nystrom S. Evidence for age-dependent in vivo conformational rearrangement within Abeta amyloid deposits. ACS Chem Biol 8, 1128–1133, doi:10.1021/cb4000376 (2013). 23521783

[35] Rasmussen J. Amyloid polymorphisms constitute distinct clouds of conformational variants in different etiological subtypes of Alzheimer’s disease. Proc Natl Acad Sci U S A 114, 13018–13023, doi:10.1073/pnas.1713215114 (2017). 29158413 PMC5724274

[36] Michno W. Multimodal Chemical Imaging of Amyloid Plaque Polymorphism Reveals Abeta Aggregation Dependent Anionic Lipid Accumulations and Metabolism. Anal Chem 90, 8130–8138, doi:10.1021/acs.analchem.8b01361 (2018). 29856605

[37] Dickson T. C. & Vickers J. C. The morphological phenotype of beta-amyloid plaques and associated neuritic changes in Alzheimer’s disease. Neuroscience 105, 99–107, doi:10.1016/s0306-4522(01)00169-5 (2001). 11483304

[38] Spires T. L. Dendritic spine abnormalities in amyloid precursor protein transgenic mice demonstrated by gene transfer and intravital multiphoton microscopy. J Neurosci 25, 7278–7287, doi:10.1523/JNEUROSCI.1879-05.2005 (2005). 16079410 PMC1820616

[39] Schmidt M. L., Robinson K. A., Lee V. M. & Trojanowski J. Q. Chemical and immunological heterogeneity of fibrillar amyloid in plaques of Alzheimer’s disease and Down’s syndrome brains revealed by confocal microscopy. Am J Pathol 147, 503–515 (1995). 7639340 PMC1869826

[40] Dickson D. W. Identification of normal and pathological aging in prospectively studied nondemented elderly humans. Neurobiol Aging 13, 179–189, doi:10.1016/0197-4580(92)90027-u (1992). 1311804

[41] Malek-Ahmadi M., Perez S. E., Chen K. & Mufson E. J. Neuritic and Diffuse Plaque Associations with Memory in Non-Cognitively Impaired Elderly. J Alzheimers Dis 53, 1641–1652, doi:10.3233/JAD-160365 (2016). 27540968 PMC6314669

[42] Rijal Upadhaya A. Biochemical stages of amyloid-beta peptide aggregation and accumulation in the human brain and their association with symptomatic and pathologically preclinical Alzheimer’s disease. Brain 137, 887–903, doi:10.1093/brain/awt362 (2014). 24519982

[43] Serrano-Pozo A., Betensky R. A., Frosch M. P. & Hyman B. T. Plaque-Associated Local Toxicity Increases over the Clinical Course of Alzheimer Disease. Am J Pathol 186, 375–384, doi:10.1016/j.ajpath.2015.10.010 (2016). 26687817 PMC4729270

[44] Murray M. E. & Dickson D. W. Is pathological aging a successful resistance against amyloid-beta or preclinical Alzheimer’s disease? Alzheimers Res Ther 6, 24, doi:10.1186/alzrt254 (2014). 25031637 PMC4055017

[45] Parvin F. Divergent Age-Dependent Conformational Rearrangement within Abeta Amyloid Deposits in APP23, APPPS1, and App(NL-F) Mice. ACS Chem Neurosci 15, 2058–2069, doi:10.1021/acschemneuro.4c00104 (2024). 38652895 PMC11099915

[46] Huang Y. Microglia use TAM receptors to detect and engulf amyloid β plaques. Nat Immunol 22, 586–594, doi:10.1038/s41590-021-00913-5 (2021). 33859405 PMC8102389

[47] Yuan P. TREM2 Haplodeficiency in Mice and Humans Impairs the Microglia Barrier Function Leading to Decreased Amyloid Compaction and Severe Axonal Dystrophy. Neuron 90, 724–739, doi:10.1016/j.neuron.2016.05.003 (2016). 27196974 PMC4898967

[48] Venegas C. Microglia-derived ASC specks cross-seed amyloid-beta in Alzheimer’s disease. Nature 552, 355–361, doi:10.1038/nature25158 (2017). 29293211

[49] Baik S. H., Kang S., Son S. M. & Mook-Jung I. Microglia contributes to plaque growth by cell death due to uptake of amyloid beta in the brain of Alzheimer’s disease mouse model. Glia 64, 2274–2290, doi:10.1002/glia.23074 (2016). 27658617

[50] Kiani Shabestari S. Absence of microglia promotes diverse pathologies and early lethality in Alzheimer’s disease mice. Cell Rep 39, 110961, doi:10.1016/j.celrep.2022.110961 (2022). 35705056 PMC9285116

