# Supplementary material for: Isotope Encoded Spatial Biology Identifies Amyloid Plaque-Age-Dependent Structural Maturation, Synaptic Loss, and Increased Toxicity
Source: Res Sq. 2025 Jan 22:rs.3.rs-5829037. Preprint. [Version 1] doi: 10.21203/rs.3.rs-5829037/v1 (PMC11838767; doi:10.21203/rs.3.rs-5829037/v1)
Supplement: Supplement 1 [file NIHPPrs5829037v1-supplement-1.pdf]

SUPPLEMENTAL FIGURES

10 months old included for analysis

N	AOI	Centroid	FWHM	Half-max intensity	R2
1	1	4524,593	8,99	0,044	0,982
2	2	4524,725	8,803	0,041	0,991
3	3	4524,811	9,365	0,038	0,997
4	5	4523,61	9,739	0,039	0,996
5	6	4524,153	8,99	0,041	0,993
6	9	4524,892	9,001	0,04	0,994
7	10	4525,458	11,491	0,036	0,994
8	12	4524,563	10,15	0,037	0,995
9	13	4526,165	9,384	0,043	0,998
10	17	4518,465	9,193	0,044	0,995
11	18	4518,577	8,427	0,042	0,998
12	19	4518,711	9,193	0,044	0,996
13	20	4518,83	8,619	0,045	0,993
14	21	4519,175	8,618	0,044	0,979
15	22	4518,833	9,001	0,046	0,995
16	23	4520,046	8,81	0,047	0,997

18 months old included for analysis

N	AOI	Centroid	FWHM	Half-max intensity	R2
1	1	4516,912	7,278	0,051	0,988
2	2	4516,051	8,235	0,049	0,992
3	3	4516,609	7,661	0,049	0,985
4	5	4516,872	7,278	0,043	0,981
5	6	4515,42	8,044	0,046	0,996
6	7	4518,295	7,117	0,05	0,966
7	8	4518,59	8,241	0,048	0,943
8	9	4518,98	7,679	0,046	0,976
9	10	4518,386	6,742	0,05	0,963
10	11	4518,742	7,491	0,051	0,96
11	12	4518,915	6,93	0,045	0,951
12	14	4517,959	7,853	0,048	0,981
13	16	4517,696	6,895	0,051	0,981
14	17	4517,457	6,32	0,052	0,997
15	19	4514,51	7,662	0,054	0,999
16	20	4514,458	7,662	0,053	0,999
17	21	4514,496	8,236	0,05	0,999
18	24	4515,076	7,278	0,054	0,998

Table S1. MALDI MSI spectral data for Aβ1-42 signals across single plaques in 10- and 18-month-old $App^{NL\text{-}F}$ mice. FWHM – full width half maximum.

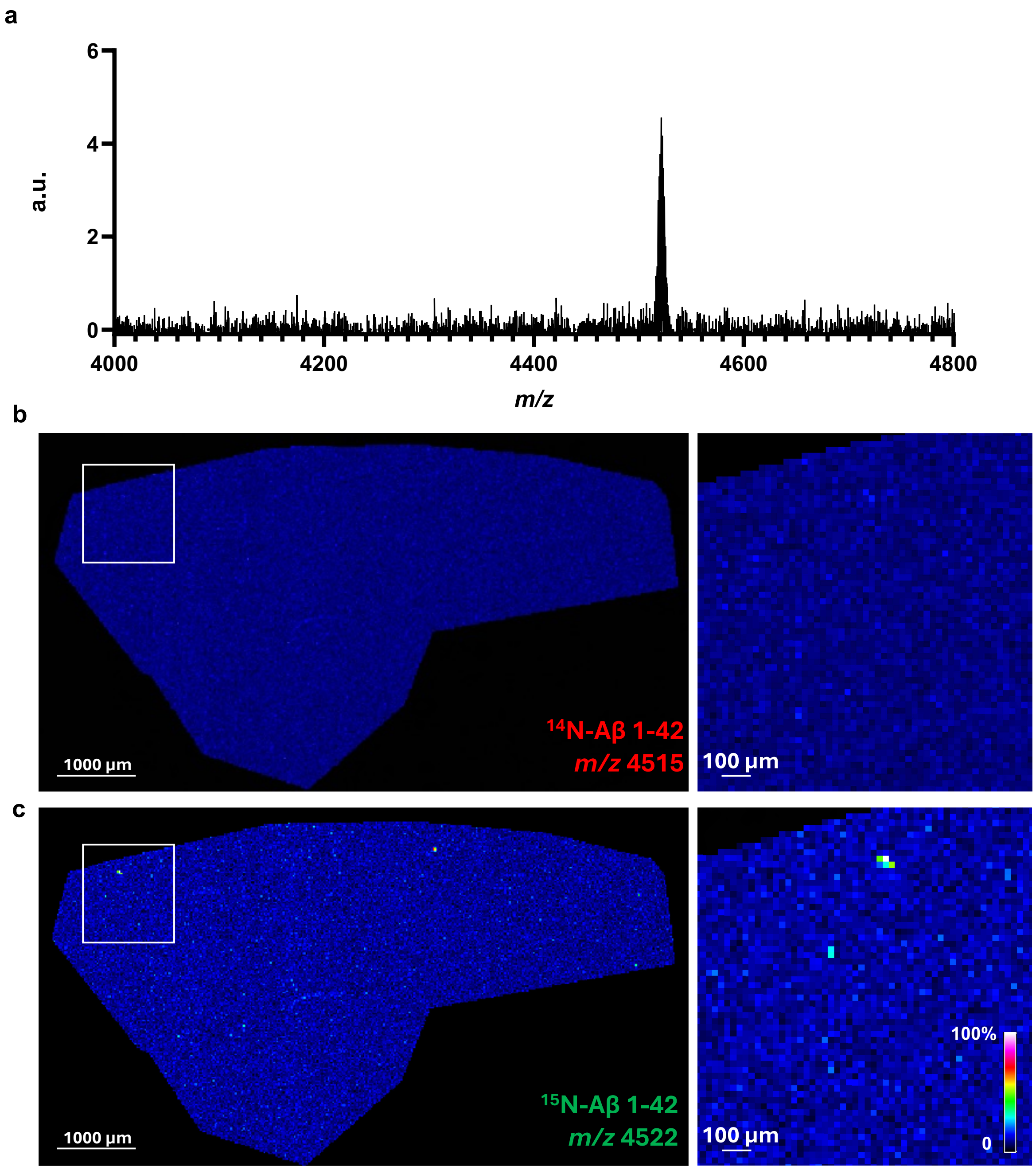

Fig S1. MALDI MSI analysis revealed that plaques in 10-month *App*$^{NL\text{-}F}$ mice contained only ^{15}N-labeled Aβ 1-42, with no unlabelled Aβ 1-42 detected. (A) The spectrum from MALDI MSI showed that the plaques in 10-month *App*$^{NL\text{-}F}$ mice contained solely Aβ 1-42. (B, C) Single ion images of Aβ 1-42 (B) showing no unlabelled (^{14}N) Aβ 1-42 (C) only ^{15}N-labeled Aβ 1-42 was detected.

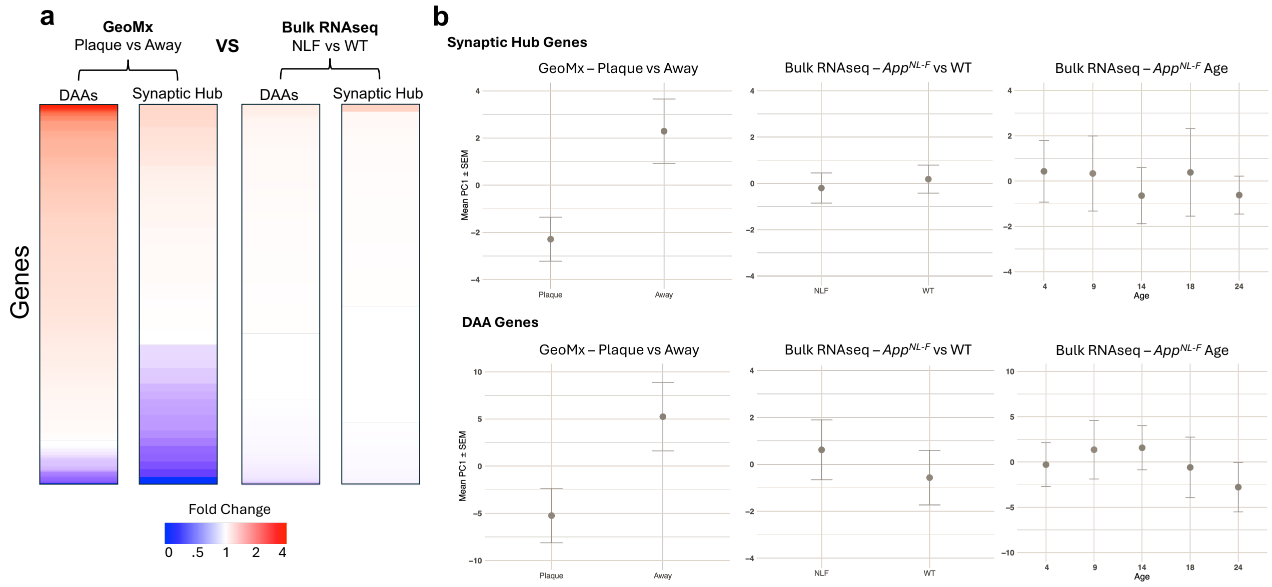

Fig S2. Amyloid induced changes in gene expression detected using either GeoMx spatial transcriptomics or Bulk RNAseq. (A) Heatmaps comparing fold change of Disease Associated Astrocytic genes (DAAs, Habib et al., 2020) and synaptic hub genes (Williams et al., 2021) by use of GeoMx spatial transcriptomic technology using an astrocytic collection in plaque vs non-plaque associated areas (n=6) or by use of bulk hippocampal RNA sequencing in 18-month-old wild-type (n=11) vs *App*$^{NL-F}$ (n=9) mice. (B) Principal Component Analysis of the DAAs and synaptic hub genes performed using data from both GeoMx spatial transcriptomics and Bulk hippocampal RNA sequencing.

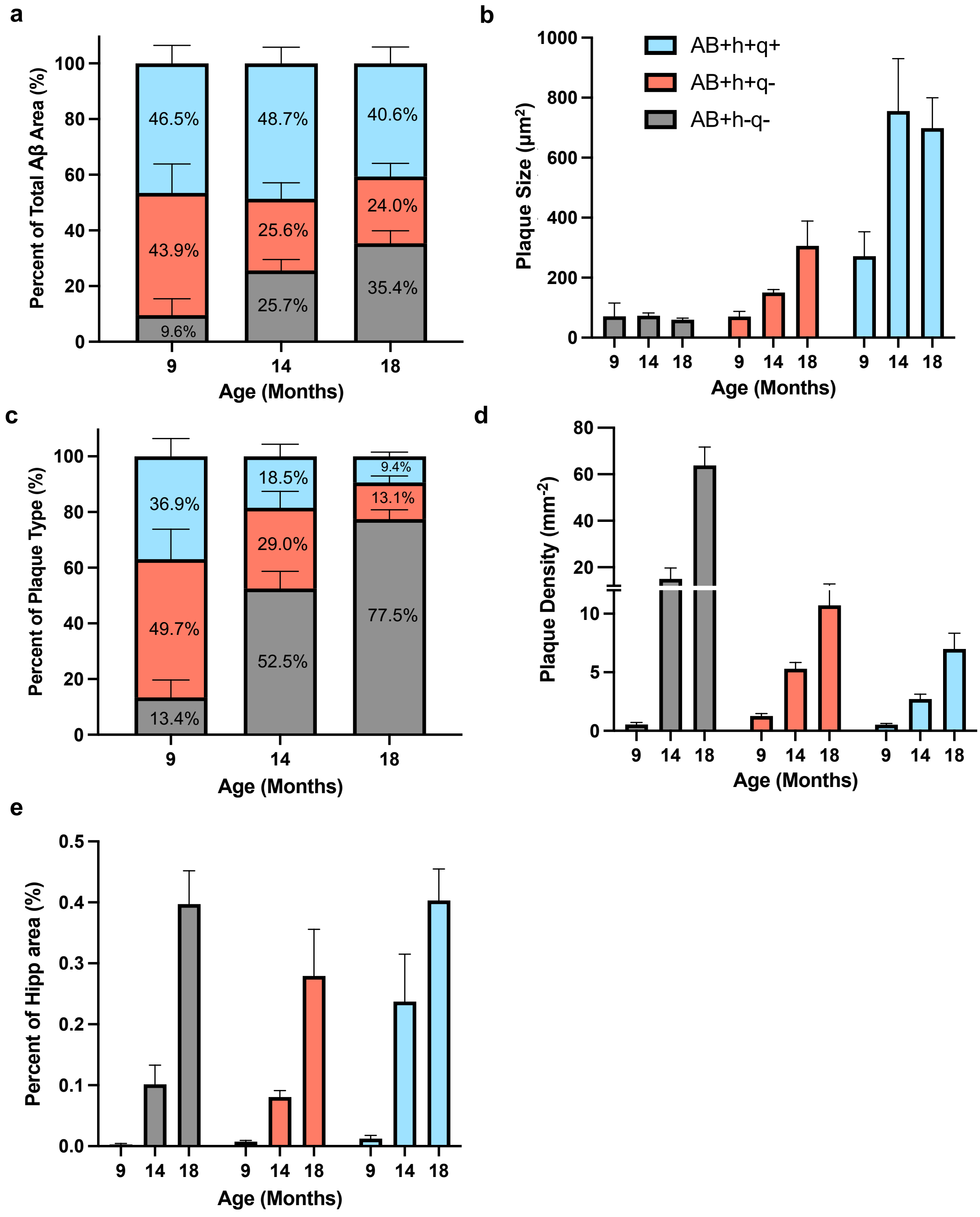

Fig S3. Plaque type characterization in 9-, 14-, and 18-month-old *App*$^{NL\text{-}F}$ mice (A) Distribution of plaque types as a percentage of the total Aβ positive area. (B) Average area occupied by each plaque type. (C) Distribution of plaque types as a percentage of the total number of Aβ plaques. (D) Plaque density of each plaque type, measured as the number of plaques per unit area in the hippocampus. (E) Percentage of the hippocampal area occupied by each plaque type. 18 months n=6 (data also shown in Figure 4), 14 months n=5, 9 months n=7 (a total of n=14 were tested, only n=7 were plaque positive in the hippocampus).
